# 18β-Glycyrrhetinic Acid Attenuates *Pasteurella multocida*-Induced Vascular Injury via Inhibition of PARP1/NF-κB p65 Nuclear Translocation

**DOI:** 10.3390/ani16172775

**Published:** 2026-09-03

**Authors:** Yuxuan Zhou, Xueping Jiang, Luyao Wang, Huabo Yan, Pu Guo, Yu Liu, Yinsheng Qiu, Jin Liu, Qirong Lu

**Affiliations:** 1 Hubei Key Laboratory of Animal Nutrition and Feed Science, School of Animal Science and Nutritional Engineering, Wuhan Polytechnic University, Wuhan 430023, China; 18799611701@163.com (Y.Z.); jxpood@163.com (X.J.); yaolu5041323@163.com (L.W.); 18290851553@163.com (H.Y.); guopu@whpu.edu.cn (P.G.); lyywfy@foxmail.com (Y.L.); qiuyinsheng6405@whpu.edu.cn (Y.Q.); 2 Wuhan Engineering and Technology Research Center of Animal Disease-Resistant Nutrition, School of Animal Science and Nutritional Engineering, Wuhan Polytechnic University, Wuhan 430023, China

**Keywords:** *P. multocida*, 18β-glycyrrhetinic acid, vascular injury, PARP1, NF-κB p65, nuclear translocation

## Abstract

*Pasteurella multocida* (Pm) is a zoonotic pathogen that causes severe hemorrhagic pneumonia in pigs, characterized by vascular injury and systemic inflammatory responses. Our previous studies demonstrated that 18β-glycyrrhetinic acid (GA), a bioactive triterpenoid derived from liquorice, protected against Pm-induced vascular inflammatory injury through suppression of poly(ADP-ribose) polymerase 1 (PARP1)-mediated nuclear factor-kappa B (NF-κB) and high mobility group box 1 (HMGB1) signaling. However, whether GA modulates PARP1-mediated NF-κB p65 nuclear translocation, a critical step in NF-κB activation, remains unclear. This study evaluated the protective effects of GA and its underlying mechanism using a Pm-infected mouse model and immunofluorescence staining of porcine iliac artery endothelial cells. Our results demonstrated that GA treatment attenuated body weight loss, ameliorated hematological and biochemical parameters, and reduced vascular structural damage in Pm-infected mice. Mechanistically, Pm infection induced PARP1 upregulation, accompanied by phospho-p65 (p-p65) nuclear translocation, all of which were suppressed by GA. PARP1 overexpression drove p-p65 nuclear accumulation, which was reversed by GA, whereas PARP1 knockdown suppressed Pm-induced p-p65 nuclear translocation. The findings demonstrated that GA protected against Pm-induced vascular injury by inhibiting PARP1-mediated NF-κB p65 nuclear translocation, supporting its potential as a candidate therapeutic agent for Pm-associated vascular injury.

## Simple Summary

*Pasteurella multocida* (Pm) is a bacterium that causes severe lung infections and injury to blood vessels in pigs, leading to significant economic losses. Current treatments rely mainly on antibiotics, but their overuse is driving the rise of drug resistance, so new alternatives are urgently needed. This study investigated whether 18β-glycyrrhetinic acid (GA), a natural compound from liquorice, could protect against Pm-induced vascular inflammatory injury and explored how it works. In infected mice, GA reduced weight loss, improved blood parameters, and protected blood vessel structure. At the cellular level, GA blocked a specific protein (PARP1) inside the nucleus that would otherwise trigger an inflammatory pathway, thereby reducing inflammation and protecting blood vessels from injury. These findings suggest that GA can be developed as an alternative agent for treating Pm infections in pigs, providing a more sustainable strategy for pig production.

## 1. Introduction

*Pasteurella multocida* (Pm) is a zoonotic pathogen that infects a broad range of hosts, including domestic and wild animals as well as humans [1,2]. Pm is classified into five capsular serogroups (A, B, D, E and F), among which serogroup A is the predominant cause of porcine pneumonia and septicemia [3,4], leading to suppurative bronchopneumonia, hemorrhagic/fibrinous pneumonia, pericarditis, peritonitis, and septicemia with fatal outcome in severe cases [5,6]. Vascular injury is widely recognized as a key pathological feature of Pm-induced hemorrhagic pneumonia, characterized by necrotizing vascular lesions, endothelial disruption, and hyperpermeability [5,7]. The vascular endothelium serves as a dynamic barrier with potent antioxidative, anti-inflammatory, and antithrombotic capacities, and its structural and functional integrity is essential for sustaining the homeostasis of various organs [8]. Endothelial dysfunction, in turn, drives the initiation and amplification of systemic inflammatory responses during infection [9,10].

In veterinary practice, antibiotics and vaccines remain the primary strategies for controlling Pm infections [11,12]. However, antibiotics fail to resolve the vascular endothelial inflammatory injury triggered by infection [13], and their overuse or misuse has exacerbated the growing challenge of antimicrobial resistance [12]. Although glucocorticoids and non-steroidal anti-inflammatory drugs effectively suppress inflammation, their clinical use is often limited by adverse effects, including gastric ulceration and immunosuppression [14]. Therefore, it is necessary to develop novel safe and effective drugs to control vascular endothelial inflammatory damage associated with Pm infection in pigs.

18β-Glycyrrhetinic acid (GA) is a pentacyclic triterpenoid derived from the root of liquorice (*Glycyrrhiza glabra* L.). It possesses a wide range of pharmacological properties, including cardiovascular protection, anti-inflammatory, anti-apoptotic, antioxidant, and immunomodulatory activities [15,16,17]. In traditional Chinese medicine, liquorice has long been recognized for its broad spectrum of therapeutic properties, including anti-inflammatory, antitussive, and detoxicant activities, and is still widely used in clinical practice [18]. Our previous study demonstrated that GA alleviated Pm-induced vascular endothelial inflammatory injury in porcine iliac artery endothelial cells (PIECs) by suppressing poly(ADP-ribose) polymerase 1 (PARP1)-mediated nuclear factor-kappa B (NF-κB) and high mobility group box 1 (HMGB1) signaling [19]. However, that study focused on protein expression in whole cells and did not examine the subcellular trafficking underlying PARP1-mediated NF-κB activation. In particular, whether GA interferes with the nuclear translocation of NF-κB p65, a critical step in NF-κB signal activation, remains unclear.

PARP1 plays a critical regulatory role in inflammatory responses and has emerged as a therapeutic target for inflammatory diseases [20,21]. Studies have demonstrated that PARP1 promotes NF-κB p65 nuclear translocation by PARylating p65 to block chromosome region maintenance 1 (Crm1)-mediated nuclear export, thereby resulting in nuclear retention of p65. In addition, PARP1 can directly interact with the p65/p50 heterodimer, serving as a transcriptional coactivator to enhance NF-κB-mediated pro-inflammatory gene expression [22]. In mouse models of lipopolysaccharide (LPS)-induced septic shock and *Salmonella enterica* serovar Typhimurium-induced colitis, PARP1 deficiency or inhibition significantly attenuated NF-κB-mediated inflammatory responses [23,24]. Our preliminary study revealed that Pm infection significantly upregulated the expression of PARP1 in PIECs and enhanced p65 phosphorylation [19]. We also found that GA treatment reduced the expression of inflammatory proteins, including PARP1, in vascular tissues of Pm-infected mice [25]. These findings point to a role for PARP1 in Pm-induced vascular inflammation, but whether this involves PARP1-mediated p65 nuclear translocation, and whether GA modulates this process, remains to be determined.

In this study, we evaluated the protective effects of GA against Pm-induced vascular inflammatory injury in mice and further elucidated its subcellular localization by examining nuclear accumulation of PARP1 and nuclear translocation of p-p65 via immunofluorescence staining. These findings may provide a theoretical basis for developing GA as a promising antibiotic alternative to prevent and treat Pm-associated vascular inflammatory injury, thereby offering a novel strategy for the clinical management of porcine pasteurellosis.

## 2. Materials and Methods

### 2.1. Bacterial Strains, Cells, and Chemicals

Pm strain HB03 (serogroups/genotypes A) was kindly provided by Prof. Bin Wu (Huazhong Agricultural University, Wuhan, China) and cultured in Tryptic Soy Agar (TSA; BD, Sparks, MD, USA) and Tryptic Soya Broth (TSB; BD) supplemented with 5% (*v*/*v*) newborn calf serum (Tianhang, Huzhou, China) at 37 °C.

PIECs were obtained from the National Collection of Authenticated Cell Cultures, Chinese Academy of Sciences, and cultured in RPMI-1640 medium (Cytiva, Marlborough, MA, USA) supplemented with 10% fetal bovine serum (Vazyme, Nanjing, China) in an incubator at 37 °C, with an atmosphere containing 5% carbon dioxide (CO_2_).

GA (CAS No.: 471-53-4) purchased from Sigma-Aldrich (St. Louis, MO, USA) was dissolved in dimethyl sulfoxide (DMSO; Sigma-Aldrich) as a stock solution and then diluted with culture medium or phosphate-buffered saline (PBS) at a ratio of 1:1000 to the desired working concentrations.

### 2.2. Animal Experiment Design

Specific pathogen-free female BALB/c mice (approximately 20 g in weight) were purchased from the Center of Laboratory Animals of Hubei Province, Wuhan, China. The mice were acclimatized in an animal room with a temperature of 22 ± 1 °C for 3 days, during which they had free access to food and water. Then, the mice were randomly divided into five groups (*n* = 10 per group): the control group, the Pm infection group, the 7.5 mg/kg body weight GA treatment group, the 15 mg/kg body weight GA treatment group, and the 30 mg/kg body weight GA treatment group. The dosages of GA were determined based on previous studies on various diseases [25,26,27,28,29,30] along with our preliminary dose-finding experiments. Except for the control group, all mice were injected intraperitoneally with 100 μL of 80 colony-forming units (CFU) of Pm HB03 to establish the infection [25]. The control group was injected with the same volume of sterile PBS. Mice in the GA-treated groups received an intramuscular injection of GA solution (7.5, 15, and 30 mg/kg body weight) 2 h prior to the infection, followed by the same dose once daily for 4 consecutive days. The control and Pm infection groups were injected with the same volume of PBS at the same time. On day 5 of the experiment, the mice were sacrificed following an overnight fast. The animal experiment was approved by the Animal Ethics Committee of Wuhan Polytechnic University (approval number WHPU202504005). To minimize suffering, mice were monitored daily for clinical signs, including appearance, posture, coat condition, mobility, food/water intake, and body weight. Humane endpoints were predefined as severe clinical signs (persistent lethargy, inability to access food/water, or a moribund state), which would trigger immediate euthanasia. No mice reached these endpoints during the study.

### 2.3. Determination of Bacterial Loads

Lung and vascular tissues were aseptically separated from the mice, weighed, homogenized, and subjected to 10-fold serial dilution in sterile PBS. The number of CFU in the samples was determined by plating serial dilutions onto TSA plates. Bacterial loads in tissues are expressed as the values of log_10_ CFU/g.

### 2.4. Hematological and Biochemical Parameters

Blood samples were collected from the mice at sacrifice to analyze hematological and biochemical parameters. For hematological parameters, the counts of white blood cells (WBC), neutrophils (Neu), lymphocytes (Lym), monocytes (Mon), eosinophils (Eos), and platelets (Plt) were determined using an ADVIA^®^2120i hematology system (Siemens, Erlangen, Germany). For biochemical parameters, heparin-anticoagulated blood samples were centrifuged at 3500 rpm for 10 min at 4 °C, and the supernatant was collected. A BS-240 automatic biochemical analyzer (Mindray, Shenzhen, China) was used to measure aspartate aminotransferase (AST), alanine aminotransferase (ALT), creatinine (CREA), urea (UREA), glucose (GLU), triglycerides (TG), total cholesterol (TC), creatine kinase (CK), and lactate dehydrogenase (LDH).

### 2.5. Histopathological Analysis

Vascular tissues harvested from mice were promptly fixed in 4% paraformaldehyde (Servicebio, Wuhan, China), dehydrated, embedded in paraffin, and cut into 5-μm-thick sections. Following hematoxylin and eosin (H&E) staining, histopathological changes were observed under an upright microscope (Olympus BX43, Tokyo, Japan) with an imaging system (Nikon, Tokyo, Japan).

### 2.6. Immunofluorescence Staining

PIECs grown to confluence on coverslips in 24-well plates were divided into five groups: a control group; a Pm infection group; and three Pm infection groups pretreated with 10, 20, or 40 µM GA. The GA concentrations were chosen based on our previous cytotoxicity assay in PIECs [19], which confirmed that these concentrations were non-cytotoxic. GA pretreatment lasted for 2 h, after which the cells were infected with Pm HB03 at a multiplicity of infection (MOI) of 100 for 10 h. Then, the cells were fixed with 4% paraformaldehyde, permeabilized with 0.5% Triton X-100, and incubated with 5% bovine serum albumin (BSA) to block nonspecific protein binding. Subsequently, the cells were co-incubated with primary antibodies against PARP1 (Proteintech, Wuhan, China) and phospho-NF-κB p65 (p-p65; Thermo Fisher Scientific, Waltham, MA, USA) overnight at 4 °C. After washing, the cells were incubated with Alexa Fluor^®^ 488- and Alexa Fluor^®^ 594-conjugated secondary antibodies (Abcam, Cambridge, UK) and counterstained with DAPI (Beyotime, Shanghai, China). The coverslips were then mounted onto slides and visualized under a laser scanning confocal microscope (Leica, Wetzlar, Germany). The fluorescence intensity was quantified by ImageJ software (version 1.54g).

### 2.7. PARP1 and p-p65 Expression and Localization in PARP1-Modulated PIECs

To investigate the role of PARP1 in GA-mediated protection against Pm infection, PARP1-overexpressing and PARP1-knockdown PIECs were generated by transient transfection with pcDNA3.0-PARP1 or PARP1-specific small interfering RNA (siRNA; GenePharma, Shanghai, China) using the Lipo8000™ transfection reagent (Beyotime), as described in our previous study [19]. Cells transfected with the empty vector pcDNA3.0 or negative control siRNA (GenePharma) served as the corresponding negative controls.

The PARP1-overexpressing cells were treated with 10, 20, or 40 µM GA for 12 h. Untreated PARP1-overexpressing cells and pcDNA3.0-transfected cells served as the untreated control and vector control, respectively. Subsequently, immunofluorescence staining for p-p65 and PARP1 was performed as described in Section 2.6 to evaluate their expression and subcellular localization.

To evaluate the effect of PARP1 silencing on Pm-induced inflammatory signaling, PARP1-knockdown cells and negative control siRNA-transfected cells were challenged with Pm at a MOI of 100 for 10 h. Untreated PARP1-knockdown cells and negative control siRNA-transfected cells served as controls. Subsequently, the expression and subcellular localization of p-p65 and PARP1 were assessed by immunofluorescence staining.

### 2.8. Statistical Analysis

Statistical analysis was performed using GraphPad Prism 10 (GraphPad Software, San Diego, CA, USA). Data are presented as mean ± standard deviation. An unpaired Student’s *t*-test was used for comparisons between two groups. For multiple comparisons, the body weight data were analyzed by two-way repeated-measures analysis of variance (ANOVA) with treatment and time as the main factors, followed by Dunnett’s post hoc test against the Pm infection group. The hematological and biochemical parameters and fluorescence intensity in Pm-infected IPECs were analyzed by one-way ANOVA, with Dunnett’s test against the Pm infection group; fluorescence intensity in PARP1-overexpressing cells (with or without GA treatment) was analyzed by one-way ANOVA, with Dunnett’s test against the PARP1-overexpressing group. *p* < 0.05 and *p* < 0.01 were considered to indicate statistically significant differences.

## 3. Results

### 3.1. The Effect of GA on the Body Weight of Pm-Infected Mice

**Figure 1:**
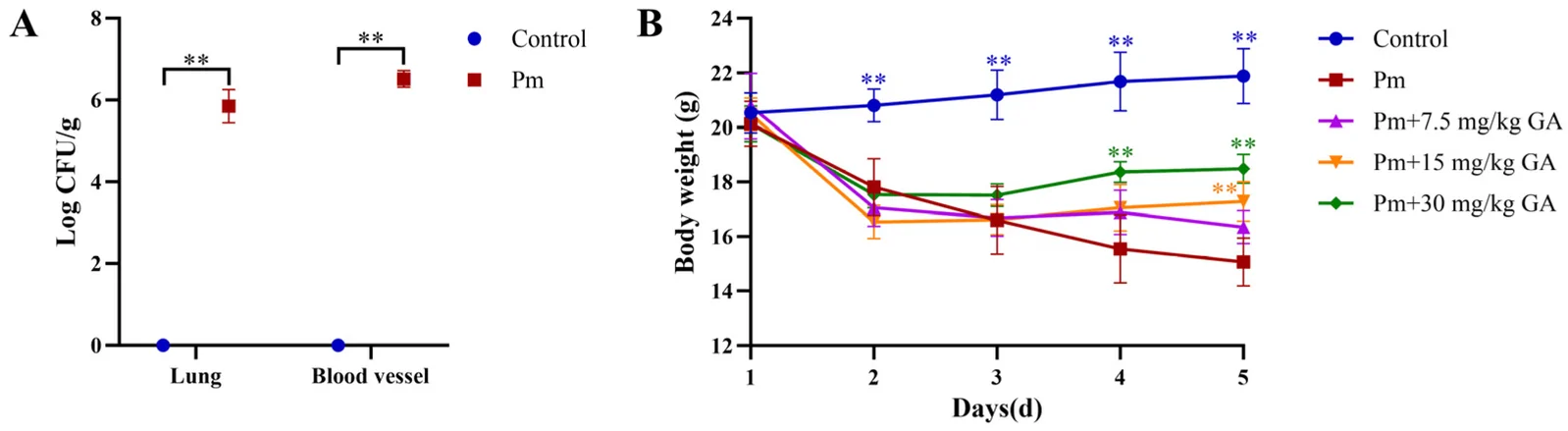
Validation of *Pasteurella multocida* (Pm) infection and the protective effect of 18β-glycyrrhetinic acid (GA) on body weight in mice. (**A**) Bacterial loads in the lung and blood vessels of mice on day 5 post-infection. ** *p* < 0.01 vs. Control. (**B**) Effect of GA on the body weight of Pm-infected mice. ** *p* < 0.01 vs. Pm group.

To validate the establishment of the Pm infection model, the bacterial loads in the lungs and vascular tissues of mice following infection were measured. Although Pm was not detected in the tissues collected from the control mice, bacterial loads in both the lungs and vascular tissues of Pm-infected mice reached approximately 10^6^ CFU/g (Figure 1A), confirming the successful establishment of the infection model. In addition, Pm-infected mice exhibited pronounced clinical signs, including lethargy, hunched posture, anorexia, and reduced locomotor activity, accompanied by a progressive loss of fur luster. GA markedly alleviated these symptoms in the treatment groups.

Meanwhile, the effect of GA on body weight changes in infected mice was evaluated. As presented in Figure 1B, the body weight of the mice gradually decreased after Pm infection (*p* < 0.01). Treatment with doses of 15 or 30 mg/kg body weight GA significantly attenuated the weight loss compared with the Pm infection group on day 5 (*p* < 0.01).

### 3.2. The Effect of GA on Pm-Induced Hematological and Biochemical Parameters

Table 1 presents the results of hematological analysis. Compared with the control group, the Pm infection group exhibited significantly elevated counts of WBC, Neu, and Mon. Compared with the Pm infection group, treatment with 30 mg/kg body weight GA significantly decreased WBC counts, while treatment with 15 or 30 mg/kg body weight GA markedly reduced Mon counts. Furthermore, Neu counts were significantly attenuated by all three GA doses. These findings indicate that GA effectively alleviates the inflammatory injury induced by Pm infection.

The plasma biochemical parameters are summarized in Table 2. Compared with the control group, the levels of AST, ALT, TC, CREA, UREA and LDH in the Pm infection group were significantly increased, while the GLU level was significantly decreased. Treatment with 15 or 30 mg/kg body weight GA significantly decreased the levels of AST, CREA, and UREA. Treatment with 30 mg/kg body weight GA also significantly reduced the ALT, TC, TG, and LDH levels. These findings suggest that Pm infection induces hepatic, renal and myocardial injury, all of which are significantly attenuated by GA.

### 3.3. The Effect of GA on Pm-Induced Vascular Histopathology

**Figure 2:**
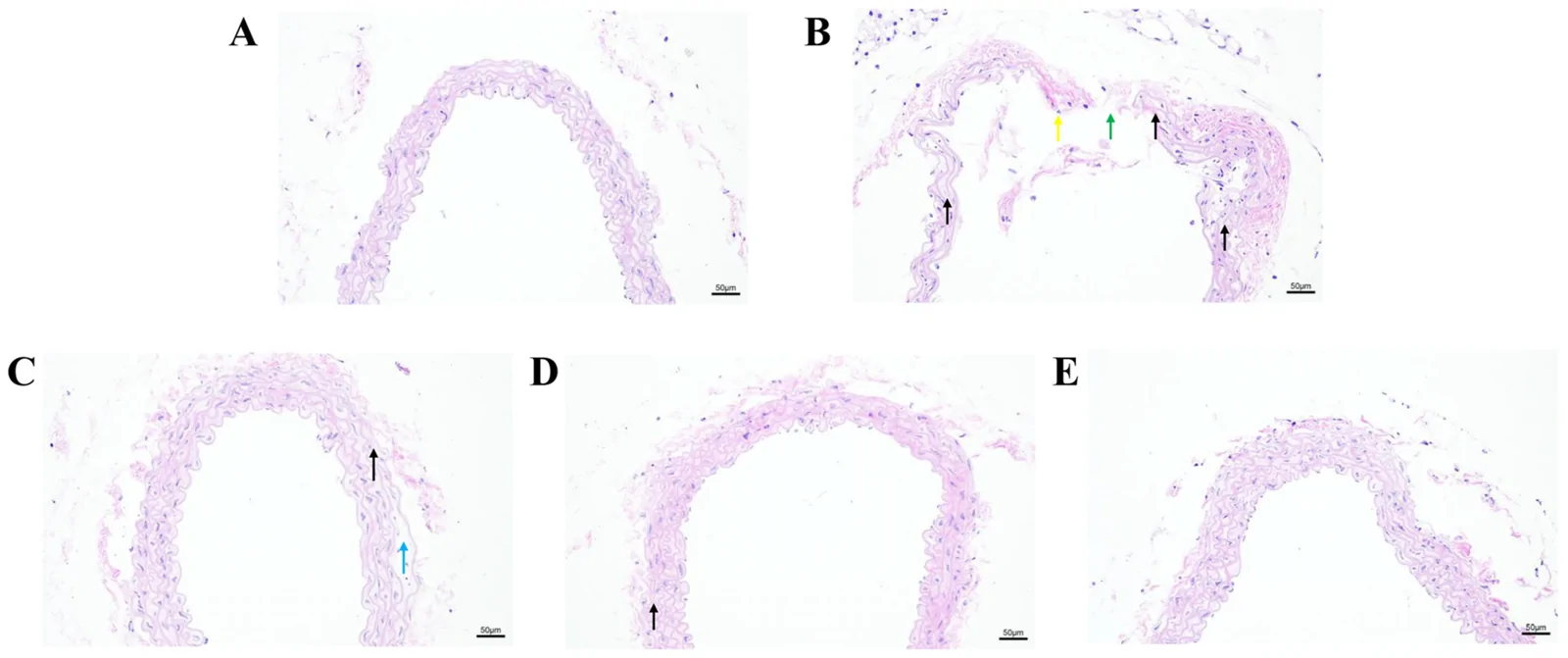
The effect of GA on vascular pathological changes in Pm-infected mice. (**A**) Control group; (**B**) Pm infection group; (**C**) 7.5 mg/kg body weight GA treatment group; (**D**) 15 mg/kg body weight GA treatment group; (**E**) 30 mg/kg body weight GA treatment group. The arrows indicate pathological changes: The green arrow indicates focal disruption of the vascular intima; the black arrow indicates disorganized elastic fibers with fragmentation; the blue arrow indicates loose elastic fibers with increased interstitial spaces; and the yellow arrow indicates inflammatory cell infiltration. Bar = 50 μm.

We evaluated the effect of GA on Pm-induced vascular injury by examining histopathological changes in vascular tissues via H&E staining (Figure 2). The control group displayed a well-defined vascular architecture with intact elastic fibers and a continuous intima (Figure 2A). In contrast, the Pm infection group exhibited disordered and severely broken elastic fibers (Figure 2B, black arrow), focal disruption of the vascular intima (Figure 2B, green arrow), and inflammatory cell infiltration (Figure 2B, yellow arrow). The 7.5 mg/kg body weight GA treatment group showed mild elastic fiber damage, including disorganized elastic fibers with focal fragmentation (Figure 2C, black arrow) and loose elastic fibers with increased interstitial spaces (Figure 2C, blue arrow). The 15 mg/kg and 30 mg/kg body weight GA treatment groups exhibited essentially normal vascular histology with clear elastic fibers and intact endothelium (Figure 2D,E). These results indicate that GA can effectively attenuate Pm-induced vascular injury in mice.

### 3.4. GA Inhibits Pm-Induced PARP-1/NF-κB p65 Nuclear Translocation

**Figure 3:**
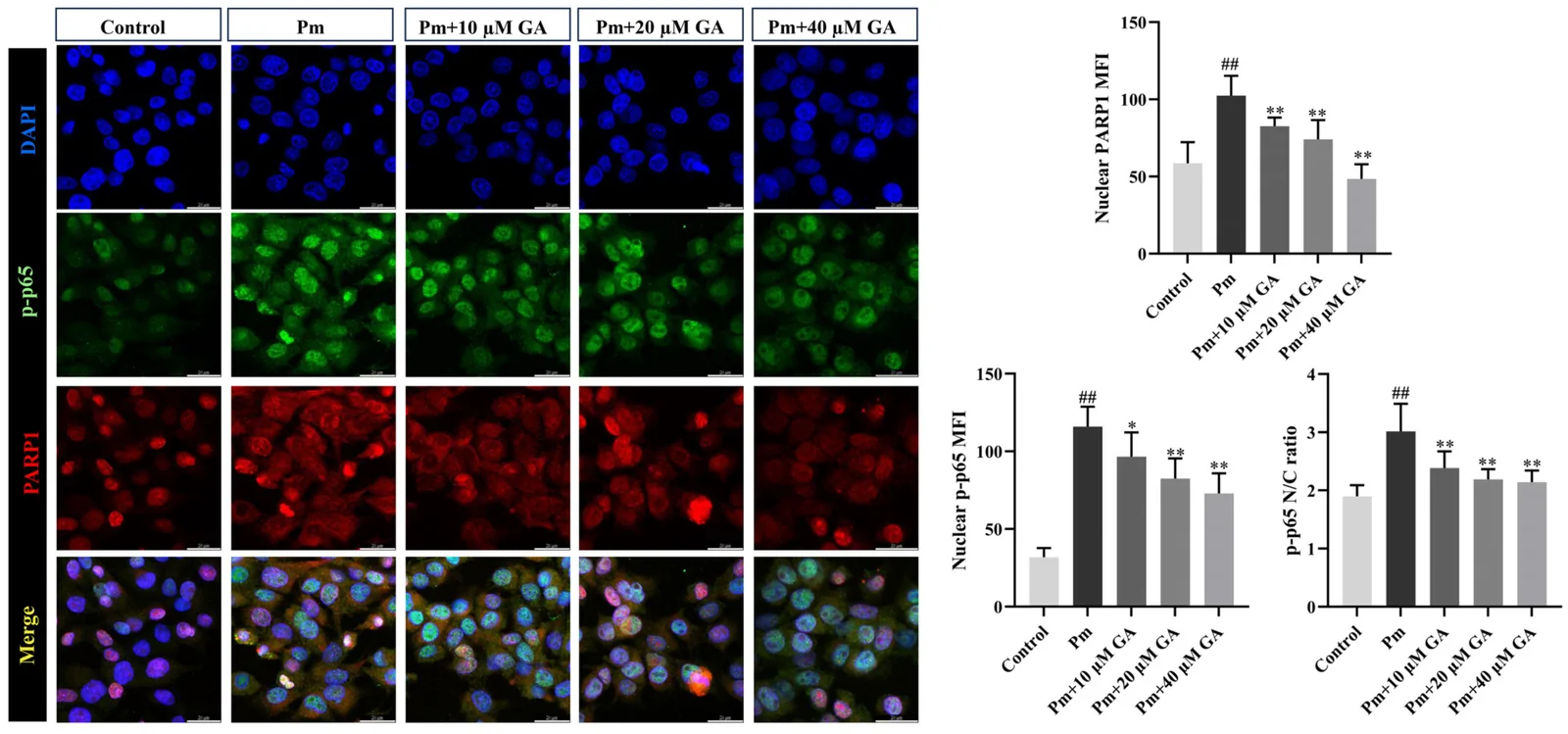
GA inhibited Pm-induced nuclear accumulation of PARP1 and nuclear translocation of p-p65 in porcine iliac artery endothelial cells (PIECs). PIECs grown on coverslips were pretreated with GA (10, 20, or 40 µM) for 2 h and then infected with Pm for 10 h. The subcellular localization of p-p65 and PARP1 were assessed using immunofluorescence staining, and the nuclear mean fluorescence intensity (MFI) of both proteins and the nucleocytoplasmic (N/C) ratio of p-p65 were quantified using ImageJ software. Control: PIECs; Pm: PIEC cells infected with Pm; Pm + 10 μM GA, Pm + 20 μM GA, and Pm + 40 μM GA: PIEC cells pretreated with 10, 20, or 40 µM GA, followed by infection with Pm. Data are presented as mean ± SD. ^##^ *p* < 0.01 vs. Control, * *p* < 0.05 vs. Pm, ** *p* < 0.01 vs. Pm. Bar = 20 μm.

Immunofluorescence staining was performed to investigate the effect of GA on the subcellular localization of PARP1 and p-p65 in Pm-infected PIECs (Figure 3). In the control group, PARP1 was predominantly localized in the nucleus, with minimal p-p65 staining in the same compartment. Upon Pm infection, the nuclear mean fluorescence intensity (MFI) of both PARP1 and p-p65 was significantly increased compared with the control group (*p* < 0.01). Similarly, the nucleocytoplasmic (N/C) ratio of p-p65 was also markedly elevated upon Pm infection (*p* < 0.01), indicating its nuclear translocation. Pretreatment with GA (10–40 μM) significantly reduced both the nuclear MFI of PARP1 and p-p65 and the N/C ratio of p-p65 (*p* < 0.05 or *p* < 0.01). Thus, GA effectively inhibited Pm-induced nuclear accumulation of PARP1 and nuclear translocation of p-p65. This finding indicates that PARP1 is critically involved in NF-κB activation triggered by Pm infection in PIECs, and that GA interferes with this signaling cascade, at least in part, by targeting PARP1.

**Figure 4:**
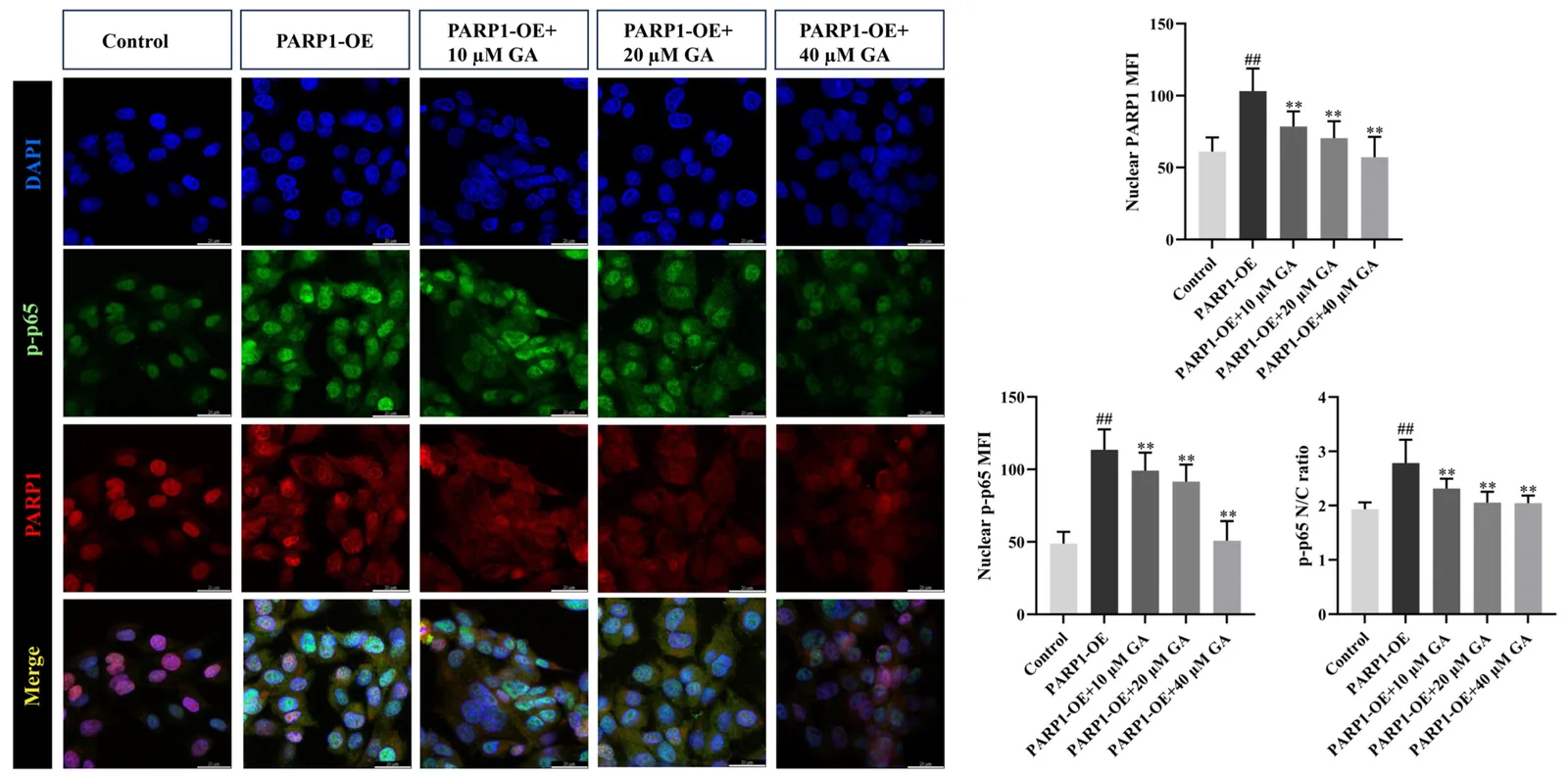
GA reversed PARP1 overexpression-induced nuclear accumulation of PARP1 and nuclear translocation of p-p65 in PIECs. The PARP1-overexpressing cells were treated with 10, 20, or 40 µM GA for 12 h. The subcellular localization of p-p65 and PARP1 were assessed using immunofluorescence staining, and the nuclear MFI of both proteins and the N/C ratio of p-p65 were quantified using ImageJ software. Control: PIECs transfected with the empty vector pcDNA3.0; PARP1-OE: PARP1-overexpressing cells; PARP1-OE + 10 μM GA, PARP1-OE + 20 μM GA, and PARP1-OE + 40 μM GA: PARP1-overexpressing cells treated with 10, 20, or 40 µM GA. Data are presented as mean ± SD. ^##^ *p* < 0.01 vs. Control., ** *p* < 0.01 vs. PARP1-OE. Bar = 20 μm.

To further validate the involvement of PARP1 as a key target of GA, PARP1 was overexpressed in PIECs to recapitulate the effects of Pm infection. Compared with empty vector controls, PARP1-overexpressing cells exhibited significantly increased MFI of both PARP1 and p-p65, as well as an elevated N/C ratio of p-p65 (*p* < 0.01) (Figure 4). These results suggest that PARP1 overexpression *per se* is sufficient to drive p-p65 nuclear translocation. Notably, treatment with GA (10–40 μM) significantly reduced the nuclear MFI of both PARP1 and p-p65 and decreased the N/C ratio of p-p65 *p* < 0.01) (Figure 4), indicating that PARP1 is a functional target of GA in vascular endothelial inflammation.

**Figure 5:**
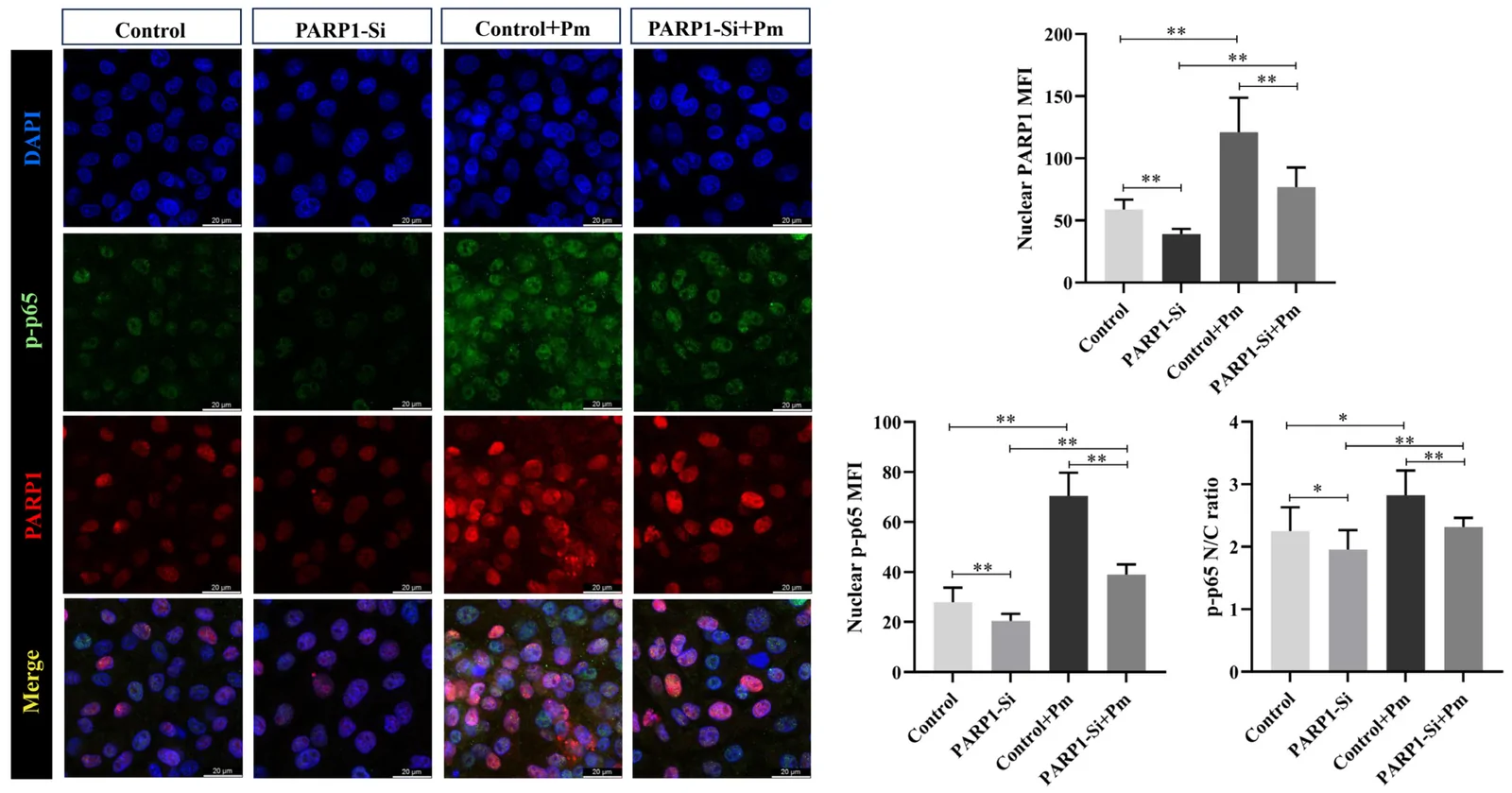
PARP1 knockdown suppressed Pm-induced nuclear accumulation of PARP1 and nuclear translocation of p-p65 in PIECs. PIECs were transfected with control siRNA or PARP1-specific siRNA and then infected with Pm for 10 h. The subcellular localization of p-p65 and PARP1 were assessed using immunofluorescence staining, and the nuclear MFI of both proteins and the N/C ratio of p-p65 were quantified using ImageJ software. Control: PIECs transfected with negative control siRNA; PARP1-Si: siRNA-mediated PARP1-knockdown cells; Control + Pm: PIEC cells transfected with negative control siRNA infected with Pm; PARP1-Si + Pm: PARP1-knockdown cells infected with Pm. Data are presented as mean ± SD. * *p* < 0.05, or ** *p* < 0.01 represents a significant difference between the groups. Bar = 20 μm.

To corroborate these findings and further confirm the role of PARP1 in Pm-induced vascular endothelial inflammatory injury, PARP1 knockdown was performed to simulate the pharmacological effects of GA in PIECs. In control siRNA-transfected cells, Pm infection markedly increased the MFI of both PARP1 and p-p65 (*p* < 0.01), as well as the N/C ratio of p-p65 (*p* < 0.05), compared with uninfected controls (Figure 5). In contrast, PARP1 knockdown largely blunted the infection-induced responses (*p* < 0.01), suggesting that PARP1 is required for Pm-induced p-p65 nuclear translocation.

## 4. Discussion

Pm infection causes hemorrhagic pneumonia characterized by necrotizing vascular lesions and severe endothelial injury in pigs [5]. Traditional antibiotic treatment fails to resolve the vascular inflammatory injury triggered by infection, and their widespread use has exacerbated the problem of antimicrobial resistance [12,13]. Natural products, owing to their diverse origins and multi-targeting properties, have attracted growing interest as potential anti-inflammatory agents [31]. Therefore, investigating the vascular inflammatory injury caused by Pm infection and the regulatory effects of natural products is of considerable importance for the development of effective therapeutic strategies. In the present study, we evaluated the protective effects of GA against Pm-induced vascular inflammatory injury in a mouse model and further investigated its mechanism by examining PARP1-mediated p-p65 nuclear translocation in PIECs. Our in vivo data showed that GA treatment significantly alleviated Pm-induced body weight loss, normalized the hematological and biochemical parameters, and reduced vascular histopathological damage. In cellular experiments, GA suppressed Pm- and PARP1 overexpression-induced nuclear accumulation of PARP1 and nuclear translocation of p-p65. In addition, the results of PARP1 knockdown experiment indicate that PARP1 is required for Pm-driven p-p65 nuclear translocation.

In this study, the lungs and vascular tissues of Pm-infected mice had bacterial loads of approximately 6 log_10_CFU/g, confirming successful infection and active bacterial dissemination [32]. The clinical signs observed in Pm-infected mice, including lethargy, hunched posture, and anorexia, match those described in earlier studies [33,34]. Moreover, Pm-infected mice showed a significant decrease in body weight compared with control mice, consistent with previous findings that Pm infection reduces weight gain in mice [7]. Notably, GA treatment significantly alleviated the clinical signs and attenuated body weight loss, suggesting that GA exerts protective effects against Pm infection. Hematological analysis showed that Pm infection significantly increased the counts of WBC, Neu, and Mon, all of which are typical indicators of systemic inflammation during bacterial infection [35]. Biochemical parameters including liver enzymes (ALT, AST, and ALP) and renal markers (CREA and UREA) are widely employed as indicators of hepatic and renal injury, and significant elevations have been reported in Pm-infected mice [36,37]. Consistently, our plasma biochemical data showed that Pm infection led to hepatic and renal dysfunction in mice. Notably, GA treatment ameliorated the Pm-induced hematological and biochemical abnormalities, suggesting that GA attenuates the systemic inflammatory response and multi-organ dysfunction triggered by Pm infection.

Bacterial infection triggers a series of local inflammatory responses, including congestion, edema, and hemorrhage at the site of infection [38]. Consistent with previous observations [34,39], Pm-challenged mice displayed pulmonary congestion, vasodilation, hemorrhage, and leukocyte recruitment. Moreover, histological examination further revealed prominent inflammatory cell infiltration in the alveolar walls, together with capillary dilation, congestion, and hemorrhage [39,40]. Vascular histopathological analysis in the present study revealed that Pm infection induced disruption of elastic fibers and intimal rupture, accompanied by inflammatory cell infiltration, indicating severe vascular damage. In our previous study, we demonstrated that Pm infection significantly upregulated PARP1, HMGB1, interleukin 1beta (IL-1β), and IL-18 protein expression in mouse vascular tissues, and that GA treatment effectively reduced the levels of these inflammatory mediators [25]. The vascular pathological alterations were effectively attenuated by GA treatment, suggesting that GA protects vascular structural integrity, likely through suppression of the local inflammatory response.

NF-κB is a master transcription factor that regulates the expression of multiple pro-inflammatory genes, and its sustained activation has been linked to various inflammatory diseases [41,42]. Because p65 nuclear translocation is critical for NF-κB activation, and PARP1 has been shown to regulate this process [43,44], we examined whether Pm infection and GA treatment affect PARP1-mediated p65 nuclear translocation in PIECs. Pm infection triggered PARP1 upregulation and p-p65 nuclear translocation in PIECs, suggesting that PARP1 is potentially involved in facilitating NF-κB activation upon bacterial infection. To further define the role of PARP1 in this process, we performed both gain-of-function and loss-of-function experiments. When PARP1 was overexpressed, p-p65 accumulated in the nucleus even in the absence of Pm, whereas PARP1 knockdown markedly reduced Pm-induced p65 nuclear translocation. Together, these observations indicate that PARP1 is required for p65 nuclear translocation upon Pm infection. Treatment with GA attenuated the nuclear MFI of both PARP1 and p-p65 in Pm-infected cells, and reversed the nuclear translocation of p-p65 driven by PARP1 overexpression. These effects are in line with earlier reports showing that loss or inhibition of PARP1 impairs p65 nuclear accumulation [43,45,46]. Notably, the observation that GA reduced PARP1 overexpression-induced p-p65 nuclear accumulation, coupled with the finding that PARP1 knockdown phenocopied the effects of GA, strongly suggests that PARP1 is a functional target through which GA interferes with NF-κB signaling. Importantly, these effects were observed at GA concentrations (10–40 μM) that do not exert direct antibacterial activity [19], supporting a PARP1-dependent mechanism. A previous study has shown that PARP1 can PARylate p65 to block its nuclear export, thereby promoting p65 nuclear accumulation [22]. This may provide a potential mechanistic basis for our observation that PARP1 is required for p65 nuclear translocation during Pm infection, a prerequisite for NF-κB-dependent transcription. Thus, GA-mediated reduction of p65 nuclear accumulation likely reflects its interference with PARP1/NF-κB signaling. This is consistent with our earlier finding that GA alleviates Pm-induced vascular endothelial inflammation through PARP1-mediated NF-κB signaling [19] and supports the notion that PARP1 is a key target through which GA dampens NF-κB activation. In addition, whether GA acts on other PARP family members and how the PARP1/NF-κB axis is regulated during bacterial infection require further investigation. It is worth noting that while immunofluorescence provides visual evidence for the subcellular localization of p-p65, nuclear-cytoplasmic fractionation combined with Western blotting would provide complementary quantitative evidence for p-p65 nuclear translocation. This represents a valuable direction for future mechanistic studies.

Given the anti-inflammatory effects of GA observed in the present study, its potential application in swine production is worth considering. Recent studies have demonstrated that dietary GA supplementation improved intestinal function and the gut microbiota, and alleviated oxidative stress and inflammatory responses in weaned piglets [47,48]. However, several translational challenges remain to be addressed. Its relatively low solubility results in poor bioavailability, limiting its clinical applications [49,50]. Although formulation strategies such as solid dispersions have been shown to improve the dissolution and bioavailability of GA [49,51], systematic pharmacokinetic data in pigs, including absorption, tissue distribution, metabolism, and elimination, are still lacking. Moreover, as GA is a natural compound with potential use in food-producing animals, regulatory considerations regarding its safety, maximum residue limits, and withdrawal periods would need to be established before practical application. Future studies are warranted to address these gaps and to evaluate the translational feasibility of GA-based interventions in veterinary practice.

## 5. Conclusions

In summary, the present study demonstrated that GA protected against Pm-induced vascular inflammatory injury in a mouse model. Mechanistically, GA suppressed PARP1-mediated NF-κB p65 nuclear translocation, thereby dampening NF-κB signaling activation. Our findings provide useful data for further development of GA as a therapeutic agent against Pm-induced vascular injury in swine production.

## Figures and Tables

**Table 1:** The effect of GA on hematological parameters in Pm-infected mice.

Parameters ^1^	Group A ^2^	Group B ^3^	Group C ^4^	Group D ^5^	Group E ^6^	*p* Value			
						Group B vs. Group A	Group B vs. Group C	Group B vs. Group D	Group B vs. Group E
WBC (10^9^/L)	3.20 ± 0.52	5.32 ± 0.51	5.19 ± 0.12	4.74 ± 0.17	3.72 ± 1.14	<0.01	0.10	0.61	<0.05
Neu (10^9^/L)	0.47 ± 0.11	2.65 ± 0.92	1.07 ± 0.41	1.11 ± 0.53	1.15 ± 0.82	<0.01	<0.05	<0.05	<0.05
Lym (10^9^/L)	2.76 ± 0.20	1.48 ± 0.90	1.46 ± 0.63	1.69 ± 0.97	1.81 ± 0.31	0.13	>0.10	0.99	0.93
Mon (10^9^/L)	0.15 ± 0.03	0.55 ± 0.05	0.32 ± 0.19	0.16 ± 0.14	0.14 ± 0.14	<0.01	0.14	<0.01	<0.01
Eos (10^9^/L)	0.06 ± 0.03	0.28 ± 0.25	0.23 ± 0.14	0.10 ± 0.03	0.09 ± 0.04	0.18	0.97	0.28	0.27
Plt (10^9^/L)	586.00 ± 14.73	353.33 ± 235.92	256.67 ± 113.17	354.33 ± 48.18	580.67 ± 221.40	0.26	0.86	>1.0	0.28

^1^ The hematological parameters were measured. WBC, white blood cells; Neu, neutrophils; Lym, lymphocytes; Mon, monocytes; Eos, eosinophils; Plt, platelets. ^2^ Group A, the control group. ^3^ Group B, Pm infection group. ^4^ Group C, 7.5 mg/kg body weight GA treatment group. ^5^ Group D, 15 mg/kg body weight GA treatment group. ^6^ Group E, 30 mg/kg body weight GA treatment group. Data of hematological parameters were presented as mean ± SD (*n* = 4).

**Table 2:** The effect of GA on the biochemical parameters in Pm-infected mice.

Parameters ^1^	Group A ^2^	Group B ^3^	Group C ^4^	Group D ^5^	Group E ^6^	*p* Value			
						Group B vs. Group A	Group B vs. Group C	Group B vs. Group D	Group B vs. Group E
AST (U/L)	119.35 ± 7.31	244.68 ± 97.18	221.58 ± 51.86	152.43 ± 36.19	151.75 ± 14.76	<0.05	0.92	<0.05	<0.05
ALT (U/L)	45.93 ± 6.50	97.35 ± 46.15	77.88 ± 15.62	55.00 ± 27.64	44.70 ± 5.12	<0.05	0.66	0.10	<0.05
TC (mmol/L)	2.65 ± 0.29	3.37 ± 0.23	2.83 ± 0.52	2.86 ± 0.20	2.61 ± 0.25	<0.05	0.10	0.12	<0.05
TG (mmol/L)	1.49 ± 0.27	2.13 ± 0.66	1.53 ± 0.32	1.62 ± 0.13	1.14 ± 0.20	0.07	0.10	0.18	<0.01
GLU (mg/dL)	5.58 ± 0.35	3.53 ± 1.34	3.52 ± 0.41	5.14 ± 1.45	5.33 ± 1.17	<0.05	1.00	0.14	0.09
CREA (mg/dL)	18.08 ± 0.98	24.23 ± 2.49	22.63 ± 1.59	19.33 ± 1.55	19.28 ± 0.62	<0.01	0.44	<0.05	<0.01
UREA (mg/dL)	5.11 ± 1.30	8.17 ± 1.37	7.04 ± 1.30	5.72 ± 1.03	4.63 ± 0.55	<0.01	0.47	<0.05	<0.01
CK (U/L)	792.15 ± 72.80	1121.13 ± 249.29	1079.58 ± 361.44	1218.58 ± 317.33	956.33 ± 232.28	0.28	1.00	0.96	0.79
LDH (U/L)	333.33 ± 25.10	479.43 ± 107.79	420.40 ± 52.84	409.53 ± 67.85	310.28 ± 58.19	<0.05	0.56	0.42	<0.05

^1^ The biochemical parameters were measured. AST, aspartate aminotransferase; ALT, alanine aminotransferase; TC, total cholesterol; TG, triglycerides; GLU, glucose; CREA, creatinine; UREA, urea; CK, creatine kinase; LDH, lactate dehydrogenase. ^2^ Group A, the control group. ^3^ Group B, Pm infection group. ^4^ Group C, 7.5 mg/kg body weight GA treatment group. ^5^ Group D, 15 mg/kg body weight GA treatment group. ^6^ Group E, 30 mg/kg body weight GA treatment group. Data of biochemical parameters were presented as mean ± SD (*n* = 4).

## Data Availability

The original contributions presented in this study are included in the article. Further inquiries can be directed to the corresponding authors.

## Abbreviations

The following abbreviations are used in this manuscript:

GA	18β-glycyrrhetinic acid
Pm	*Pasteurella multocida*
PARP1	Poly(ADP-ribose) polymerase 1
NF-κB	Nuclear factor-kappa B
PIECs	Porcine iliac artery endothelial cells
HMGB1	High mobility group box 1
Crm1	Chromosome region maintenance 1
WBC	White blood cells
Neu	Neutrophils
Lym	Lymphocytes
Mon	Monocytes
Eos	Eosinophils
Plt	Platelets
AST	Aspartate aminotransferase
ALT	Alanine aminotransferase
CREA	Creatinine
UREA	Urea
GLU	Glucose
TG	Triglycerides
TC	Total cholesterol
CK	Creatine kinase
LDH	Lactate dehydrogenase
MOI	Multiplicity of infection
MFI	Mean fluorescence intensity
N/C	Nucleocytoplasmic
